# Cell-Cycle Dependent Expression of a Translocation-Mediated Fusion Oncogene Mediates Checkpoint Adaptation in Rhabdomyosarcoma

**DOI:** 10.1371/journal.pgen.1004107

**Published:** 2014-01-16

**Authors:** Ken Kikuchi, Simone Hettmer, M. Imran Aslam, Joel E. Michalek, Wolfram Laub, Breelyn A. Wilky, David M. Loeb, Brian P. Rubin, Amy J. Wagers, Charles Keller

**Affiliations:** 1 Pediatric Cancer Biology Program, Papé Family Pediatric Research Institute, Department of Pediatrics, Oregon Health & Science University, Portland, Oregon, United States of America; 2 The Howard Hughes Medical Institute and Department of Stem Cell and Regenerative Biology, Harvard University, Cambridge, Massachusetts, United States of America, and Joslin Diabetes Center, Boston, Massachusetts, United States of America; 3 Department of Pediatric Oncology, Dana Farber Cancer Institute and Division of Pediatric Hematology/Oncology, Children's Hospital, Boston, Massachusetts, United States of America; 4 Department of Epidemiology and Biostatistics, University of Texas Health Science Center, San Antonio, Texas, United States of America; 5 Department of Radiation Medicine, Oregon Health & Science University, Portland, Oregon, United States of America; 6 Division of Medical Oncology, Sidney Kimmel Comprehensive Cancer Center, Johns Hopkins University, Baltimore, Maryland, United States of America; 7 Division of Pediatric Oncology, Sidney Kimmel Comprehensive Cancer Center, Johns Hopkins University, Baltimore, Maryland, United States of America; 8 Departments of Anatomic Pathology and Molecular Genetics, Taussig Cancer Center and Lerner Research Institute, Cleveland Clinic Foundation, Cleveland, Ohio, United States of America; University of Washington, United States of America

## Abstract

Rhabdomyosarcoma is the most commonly occurring soft-tissue sarcoma in childhood. Most rhabdomyosarcoma falls into one of two biologically distinct subgroups represented by alveolar or embryonal histology. The alveolar subtype harbors a translocation-mediated *PAX3:FOXO1A* fusion gene and has an extremely poor prognosis. However, tumor cells have heterogeneous expression for the fusion gene. Using a conditional genetic mouse model as well as human tumor cell lines, we show that that Pax3:Foxo1a expression is enriched in G_2_ and triggers a transcriptional program conducive to checkpoint adaptation under stress conditions such as irradiation *in vitro* and *in vivo*. Pax3:Foxo1a also tolerizes tumor cells to clinically-established chemotherapy agents and emerging molecularly-targeted agents. Thus, the surprisingly dynamic regulation of the *Pax3:Foxo1a* locus is a paradigm that has important implications for the way in which oncogenes are modeled in cancer cells.

## Author Summary

Rare childhood cancers can be paradigms from which important new principles can be discerned. The childhood muscle cancer *rhabdomyosarcoma* is no exception, having been the focus of the original 1969 description by Drs. Li and Fraumeni of a syndrome now know to be commonly caused by underlying *p53* tumor suppressor loss-of-function. In our studies using a conditional genetic mouse model of alveolar rhabdomyosarcoma in conjunction with human tumor cell lines, we have uncovered that the expression level of a translocation-mediated fusion gene, *Pax3:Foxo1a*, is dynamic and varies during the cell cycle. Our studies support that Pax3:Foxo1a facilitate the yeast-related process of checkpoint adaptation under stresses such as irradiation. The broader implication of our studies is that distal *cis* elements (promoter-influencing regions of DNA) may be critical to fully understanding the function of cancer-associated translocations.

## Introduction

Rhabdomyosarcoma (RMS) is the most common childhood soft tissue sarcoma. Historically, RMS has been thought to arise from muscle because of the expression of myogenic markers. Most childhood RMS falls into one of two biologically distinct subgroups: alveolar (aRMS) or embryonal (eRMS). aRMS is the more aggressive variant with a survival rate of less than 20% when metastatic due to chemotherapy and radiation resistance [1]. aRMS is characterized by a frequent t(2;13) chromosomal translocation, which results in the *PAX3:FOXO1A* fusion gene, or less frequently by a t(1;13) mediated *PAX7:FOXO1A* fusion oncogene [1]. Clinically, the aggressive behavior of aRMS has been attributed to PAX3:FOXO1A transcriptional reprograming because fusion negative aRMS have a more favorable outcome similar to eRMS [2], [3], [4].

We previously developed a mouse model of aRMS employing a conditional knock-in approach that expresses *Pax3:Foxo1a* from the native *Pax3* locus in fetal and postnatal myoblasts [5], [6], [7]. In this model, Pax3:Foxo1a was necessary but not sufficient for aRMS tumor initiation. Interestingly, cells expressing high levels of *Pax3:Foxo1a* were more prevalent in metastatic tumors [7]. The heterogeneity of Pax3:Foxo1a expression in primary and metastatic tumors, and enrichment in the latter, suggested that Pax3:Foxo1a might be selectively expressed in a subset of aRMS cells; alternatively, Pax3:Foxo1a expression might be temporally regulated. In the current study we present striking evidence that Pax3:Foxo1a is expressed in a dynamic manner and mediates a G_2_-specific program enabling checkpoint adaptation and refractoriness to therapy.

## Results

### Pax3:Foxo1a expression is dynamic in mouse aRMS cells

**Figure 1:**
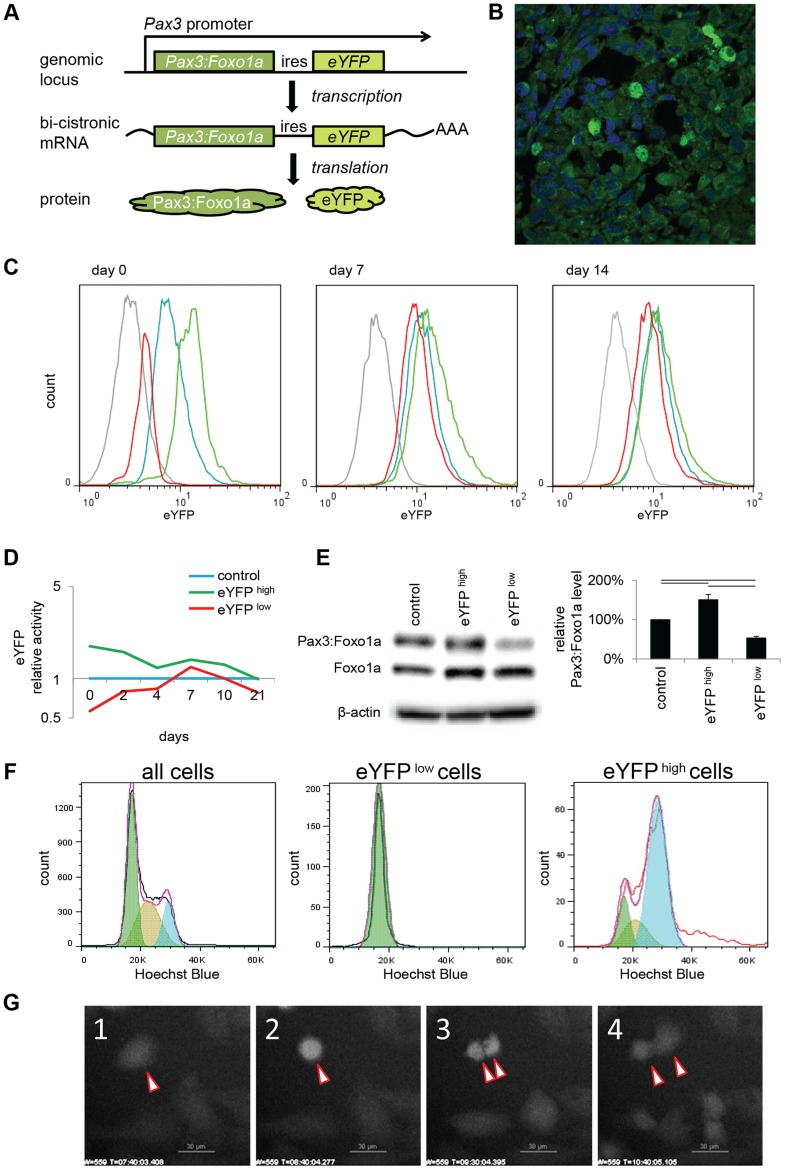
eYFP activity and Pax3:Foxo1a expression is cell cycle specific. (A) Diagrammatic representation of the conditional *Pax3:Foxo1a* knock-in allele by which *eYFP* is expressed as a second cistron on the same mRNA as *Pax3:Foxo1a* at the native *Pax3* promoter. (B) Heterogeneity of eYFP expression in a murine aRMS tumor by immunofluorescence. (C) eYFP fluorescence of eYFP sorted cells overtime as measured by FACS. Grey: C2C12 (negative control), blue: no sorted cells, green: eYFP activity high cells, red: eYFP activity low cells. (D) Mean of relative eYFP activity in panel 1C measured by FACS. (E) Western blot analysis using eYFP sorted cells. Plotted are relative protein levels of Pax3:Foxo1a/β-Actin. Mean ± SE were obtained from three independent immunoblottings. Black line shows significant difference (p<0.05). (F) eYFP activity and cell cycle analysis using Hochest33342 staining for mouse primary cell culture U23674. Green shows G_0_/G_1_ phase, brown shows S phase, and blue shows G_2_/M phase. (G) Time-lapse experiment of eYFP activity (select frames over 16 hours). See also corresponding Movie S1.

In our genetically-engineered conditional knock-in mouse model of aRMS, *e* *Y* *FP* is expressed as a second cistron on the same mRNA as *Pax3:Foxo1a* (Figure 1A). We have observed heterogeneity of eYFP expression among tumor cells *in situ* (Figure 1B). To first examine Pax3:Foxo1a expression as a function of time, we flow sorted Pax3:Foxo1a^low^ and Pax3:Foxo1a^high^ cells using eYFP signal in two independent murine aRMS primary cultures (Figure 1C and 1D; Figure S1A and S1B). Comparison of Pax3:Foxo1a protein levels for sorted populations showed Pax3:Foxo1a^low^ cells possessed much reduced levels of Pax3:Foxo1a protein (Figure 1E and Figure S1C). However, FACS analysis over time revealed that the eYFP signal of Pax3:Foxo1a^low^ and Pax3:Foxo1a^high^ tended towards the mean eYFP fluorescence intensity of unsorted tumor cells with time and/or cell divisions (Figure 1C and 1D; Figure S1A and S1B). Thus, Pax3:Foxo1a^high^ cell could dynamically reduce expression of eYFP from the *Pax3:Foxo1a* locus, and Pax3:Foxo1a^low^ cells could dynamically increase expression of eYFP from the *Pax3:Foxo1a* locus. We further confirmed that eYFP expression was indeed reflective of Pax3:Foxo1a expression in terms of protein half-life. Figure S1E and S1F shows levels of eYFP signal and Pax3:Foxo1a protein stability after translation inhibition by cycloheximide (CHX). Akin to the strong correlation between eYFP and *Pax3:Foxo1a* expression at the protein level (Figure 1 and Figure S1C), the protein half-lives of Pax3:Foxo1a and eYFP were roughly similar at 31.6 and 44.7 hours (Figure S1E and S1F), thereby affirming that eYFP is a reasonable surrogate for transcription of Pax3:Foxo1a from the *Pax3* locus (we do however acknowledge that eYFP is a better marker of the start of *Pax3:Foxo1a* transcription than the end of Pax3:Foxo1a transcription or protein expression (i.e., since *eYFP* is expressed on the same mRNA as *Pax3:Foxo1a*, the beginning of fluorescence should coincide with the initial presence of the Pax3:Foxo1a transcript). Thereafter, eYFP is susceptible to photo-bleaching and possible proteasomal degradation sooner than the 44 hours observed under conditions of cyclohexamide treatment (Figure S1F)).

### Pax3:Foxo1a expression is dynamically regulated during the cell cycle

To investigate what conditions affect the dynamic alteration of Pax3:Foxo1a expression in aRMS cells, we compared eYFP fluorescence to cell cycle phase as determined by staining with the DNA dye Hoechst33342. Almost all Pax3:Foxo1a^low^ cells existed in G_0_/G_1_ (2N) stage, while to our surprise Pax3:Foxo1a^high^ cells were G_2_/M or hyperdiploid/multinuclear (≥4N) cells (Figure 1F and Figure S1D). We next performed time-lapse experiments of eYFP activity by confocal microscopy. Figure 1G shows in time-lapse images that eYFP activity during cell division is transiently but markedly increased, particularly in pre-mitotic cells. Interestingly, the level of eYFP in some multinuclear cells remained at a high level in cells that appeared to be unable to undergo telophase/cytokinesis (Movie S1).

**Figure 2:**
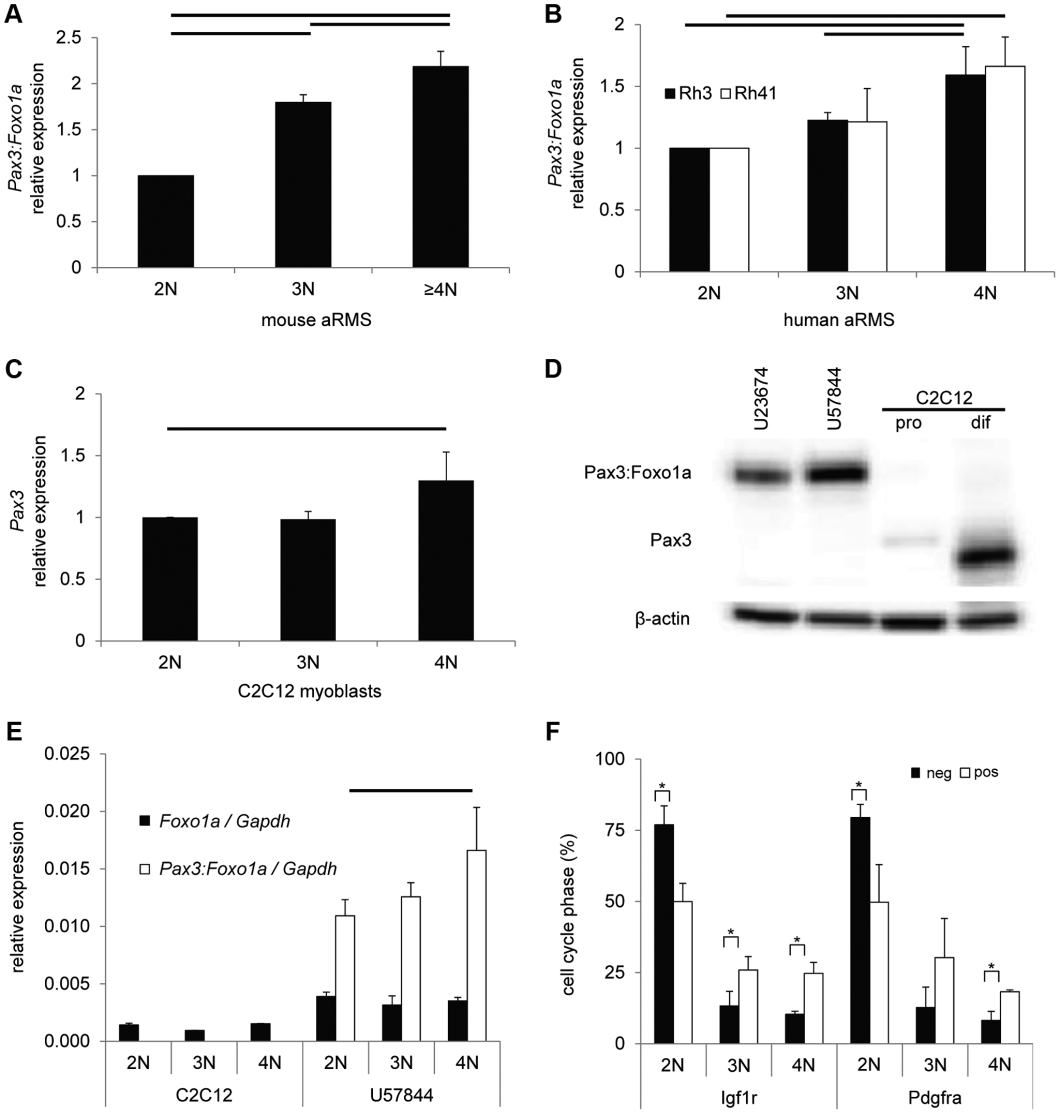
Pax3:Foxo1a activity is cell cycle dependent. (A) mRNA expression of *Pax3:Foxo1a* normalized by *Gapdh* in U23674 mouse aRMS primary cell culture sorted by DNA content. Black lines show significant differences (p<0.05). (B) mRNA expression of *PAX3:FOXO1A* normalized by *GAPDH* in Rh3 and Rh41 human aRMS cell lines sorted by DNA content. (C) mRNA expression of *Pax3* normalized by *Gapdh* in C2C12 murine myoblasts sorted by DNA content. (D) Western blot analysis of Pax3 and Pax3:Foxo1a in unsorted murine U23674 aRMS cells (genotype *Pax3* (Pax3:Foxo1a activated/Pax3:Foxo1a activated)), murine U57844 aRMS cells (genotype *Pax3* (wt/Pax3:Foxo1a activated)), proliferative C2C12 myoblasts (pro) and differentiating C2C12 myoblasts (dif). (E) mRNA expression of Foxo1 and *Pax3:Foxo1a* normalized to *Gapdh* in C2C12 myoblasts and the U57844 mouse aRMS primary cell culture. (F) Cell cycle analysis after sorting for Pax3:Foxo1a targets Igf1r or Pdgfra in mouse aRMS tumor cells. Nearly twice as many 4N cells are Igf1r (or Pdgfra) positive versus Igf1r (or Pdgfra negative), suggesting these Pax3:Foxo1a targets may have a functional role late in the cell cycle (* P<0.05). pos, positive. Neg, negative.

We next performed QPCR of *Pax3:Foxo1a* and *PAX3:FOXO1A* using cell cycle specific sorted mouse and human aRMS cells, respectively. Both mouse and human aRMS cells showed significant differences in the mRNA expression of *Pax3:Foxo1a* and *PAX3:FOXO1A* in the transition from 2N (G_1_) to 3N (S phase) and 4N (G_2_/M) cells (Figure 2A and 2B) affirming cross-species relevance of the cell cycle dependent mRNA regulation of *Pax3:Foxo1a* expression.

To investigate the transcriptional basis of this Pax3:Foxo1a dynamic expression, we performed QPCR of *Pax3* and *Foxo1* using cell cycle specific sorted C2C12 mouse myoblast cells of the genotype *Pax3*(*wt/wt*) and mouse aRMS primary tumor cells of the genotype *Pax3*(*wt/Pax3:Foxo1a*). C2C12 myoblasts showed significant increases in *Pax3* mRNA levels for 4N cells when compared with 2N cells (Figure 2C). *Pax3* was not detectable in aRMS cells at the mRNA level (data not shown), which was also reflected in the absence of expression of Pax3 protein in aRMS cells by western blotting (Figure 2D). This result is consistent with our prior studies suggesting that Pax3:Foxo1a causes decreased expression of the wildtype *Pax3* locus [5], [6]. By contrast, *Foxo1* mRNA expression did not differ between 2N and 4N in either C2C12 myoblasts or aRMS tumor cells (Figure 2E). Thus, the cell cycle dependence of *Pax3:Foxo1a* may in some part be attributable to increased *Pax3* promoter activity at G_2_/M versus G_1_ in C2C12 myoblasts, but *Pax3:Foxo1a* transcript level is so significantly increased over *Pax3* in aRMS cells that other factors related to the chromosomal fusion are likely responsible, *e.g.* gain of a *Foxo1a* 3′ *cis*-enhancer, or loss of a *Pax3* 3′ *cis*-repressor repressor. From the design of the conditional knock-in allele [5], this element(s) can be inferred to exist in the 9.3 kB of the *Foxo1a* 3′ region containing exons 2 and 3 and untranslated region (6.5 kb), or exons 8–10 of *Pax3*. We also cannot exclude that stabilization of the *Pax3:Foxo1a* transcript may to some degree play a role, and this stabilization may or may not be related to the *Foxo1a* cis-elements on the chimeric mRNA.

Because *Pdgfra* [8] and *Igf1r* [9] are well known direct downstream targets of Pax3:Foxo1a, we determined whether these targets were expressed to any degree in 4N (G_2_/M) cells. We first sorted aRMS tumor cells for Pdgfra or Igf1r positivity versus negativity, then performed DNA content analysis. For both receptor tyrosine kinases (RTKs), the majority of cells with positive RTK surface expression were 2N (Figure 2F). However, nearly twice as many 4N cells are Igf1r (or Pdgfra) positive versus Igf1r (or Pdgfra) negative, suggesting these Pax3:Foxo1a targets may have a functional role late in the cell cycle, such as the Igf1r-mediated radioresistance seen for other forms of cancer [10].

### Pax3:Foxo1a expression is specific to G_2_ and acts in G_2_/M checkpoint adaptation

**Figure 3:**
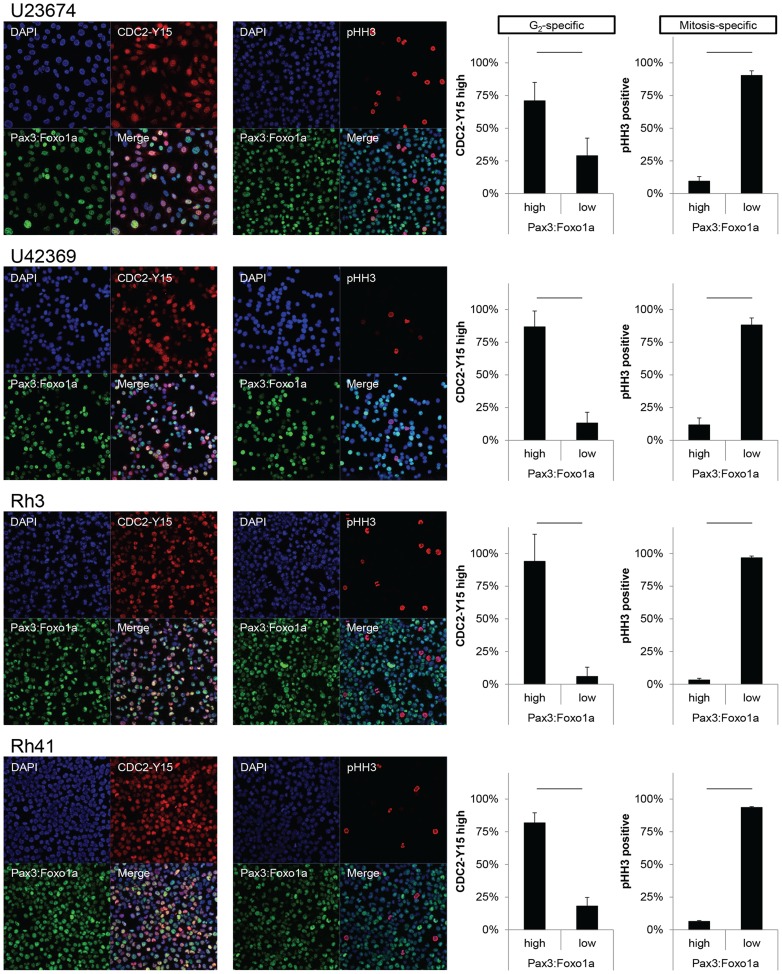
Pax3:Foxo1a is expressed in G_2_ for mouse and human aRMS. Immunocytochemistry for Pax3 (green), pHH3 (red) and DAPI (blue) or Pax3 (green), pCDC2 (red) and DAPI (blue). Numbers are relative rate of Pax3:Foxo1a high or low cells/pHH3 positive cells and Pax3:Foxo1a high or low cells/CDC2-Y15 high cells. Black line shows significant difference (p<0.05).

**Figure 4:**
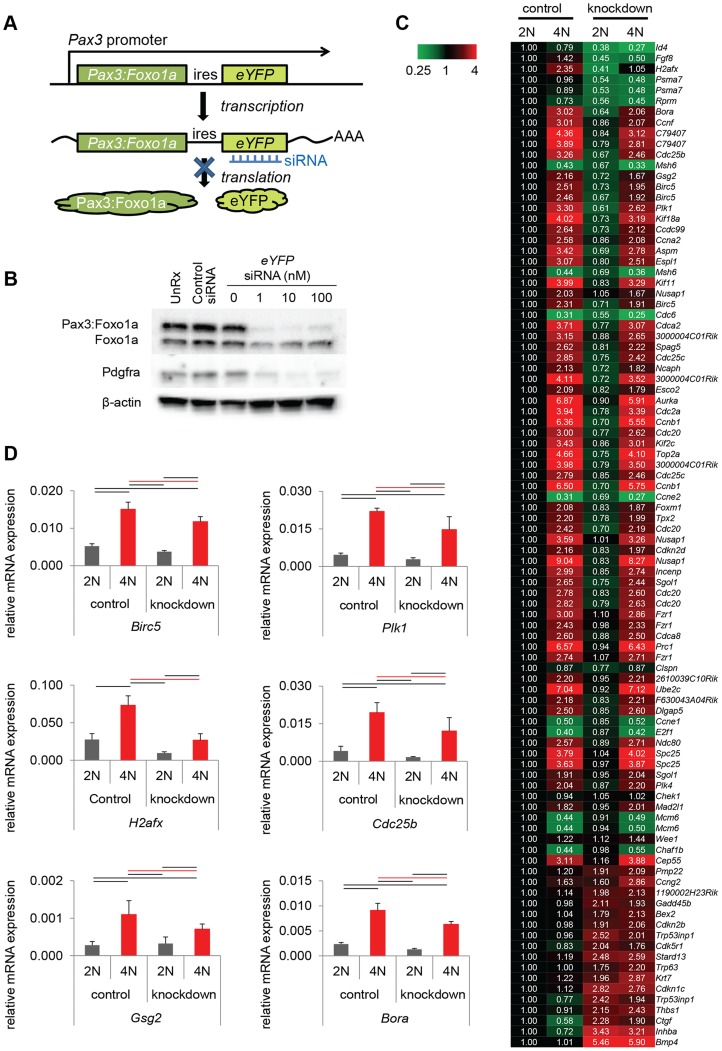
Pax3:Foxo1a induces G_2_/M checkpoint adaptation gene in G_2_/M. (A) Diagrammatic representation of *Pax3:Foxo1a* knockdown strategy using *eYFP* siRNA. (B) Knockdown of the *Pax3:Foxo1a* protein by siYFP. Total cell lysates were isolated 48 h after transfection. Pax3:Foxo1a was detected with an antibody targeting the C-terminus of Foxo1a. (C) Differential expression of 60 of cell cycle genes (as annotated by Gene Ontology) for DNA content with or without *Pax3:Foxo1a* knockdown. (D) mRNA expression by QPCR of *Plk1, Cdc25b, H2afx* and *Birc5* normalized to *Gapdh* in DNA content-sorted U23674 mouse aRMS primary tumor cells with or without *Pax3:Foxo1a* knockdown. Black and red line shows significant difference (p<0.05).

To determine the role of Pax3:Foxo1a in G_2_, M or G_2_/M checkpoint, we examined markers of each cell cycle phase under non-stress or stress conditions. Immunocytochemistry is presented in Figure 3 is a for Pax3:Foxo1a (Pax3) with phospho-histone H3 (pHH3), a marker of mitosis, or CDC2-Y15 (pCDC2), a negative marker of entry into mitosis that is commonly expressed in G_2_ (CDC2-Y15 is phosphorylated by Wee1 kinase, which then negatively regulates Cdc2 kinase [11]; CDC2-Y15 is present starting in late G_1_ then also in S, and G_2_ phases, but absent in M [12]). In murine aRMS primary cultures U23674 and U42369, pHH3 positive metaphase cells did not express Pax3:Foxo1a protein and yet most pCDC2 positive cells expressed Pax3:Foxo1a very highly (Figure 3). These results suggest that Pax3:Foxo1a is expressed in the G_2_ cell cycle phase but not M phase. Human aRMS cell lines Rh3 and Rh41 showed identical results (Figure 3). Next, we sought to understand the function of Pax3:Foxo1a in G_2_. For this purpose we performed genome-wide expression analysis using cells sorted at specific stages of the cell cycle (2N vs. 4N) with or without *Pax3:Foxo1a* siRNA knockdown (Figure 4A). Because *eYFP* is expressed as a second cistron in the targeted *Pax3:Foxo1a-ires-* *eYFP* allele, we anticipated that siRNA for *eYFP* would knock down not only eYFP but also Pax3:Foxo1a. Western blotting of Pax3:Foxo1a and native Foxo1a protein 48 hours after siRNA transfection showed that *eYFP* siRNA efficiently and specifically knocked down Pax3:Foxo1a protein (Figure 4B). Protein expression of the Pax3:Foxo1a transcriptional target Pdgfra was also reduced (Figure 4B).

From genome-wide expression analysis of 2N vs. 4N sorted cells with or without *Pax3:Foxo1a* siRNA knockdown, we found several genes implicated in the process of G_2_/M checkpoint adaptation to be down-regulated in G_2_/M (4N cells) when Pax3:Foxo1a was knocked down (Figure 4C; Table S1 shows all data analyzed by ANOVA (<0.05) using the multiple comparison correction method of Benjamini and Hochberg). Checkpoint adaptation is the process by which unicellular organisms or cancer cells progress through a delayed cell cycle checkpoint (G_2_ or by analogy the mitotic spindle assembly checkpoint) in lieu of programmed cell death, but before DNA damage is completely repaired [13], [14], [15]. Factors implicated in checkpoint adaptation are similar to those involved in checkpoint recovery (after complete repair of DNA damage), but additionally require anti-apoptotic signals [14]. Select G_2_/M checkpoint adaptation genes implicated in this experiment, the DNA damage sensing/checkpoint progression factors *Plk1*, *Cdc25b*, *H2afx* and the cell survival factor *Birc5* (*Survivin*), were validated for differential expression by QPCR (Figure 4D). Whether these genes are direct transcriptional targets of Pax3:Foxo1a was investigated by interrogating loci for reported nearby Pax3:Foxo1a binding sites [16]. Most potential regulatory sites were greater than 60 kB away (Table S2). While regulatory sequences can be hundreds of kBs away from the target gene, it remains possible that these genes may also be regulated indirectly by other Pax3:Foxo1a target genes or miRNAs.

**Figure 5:**
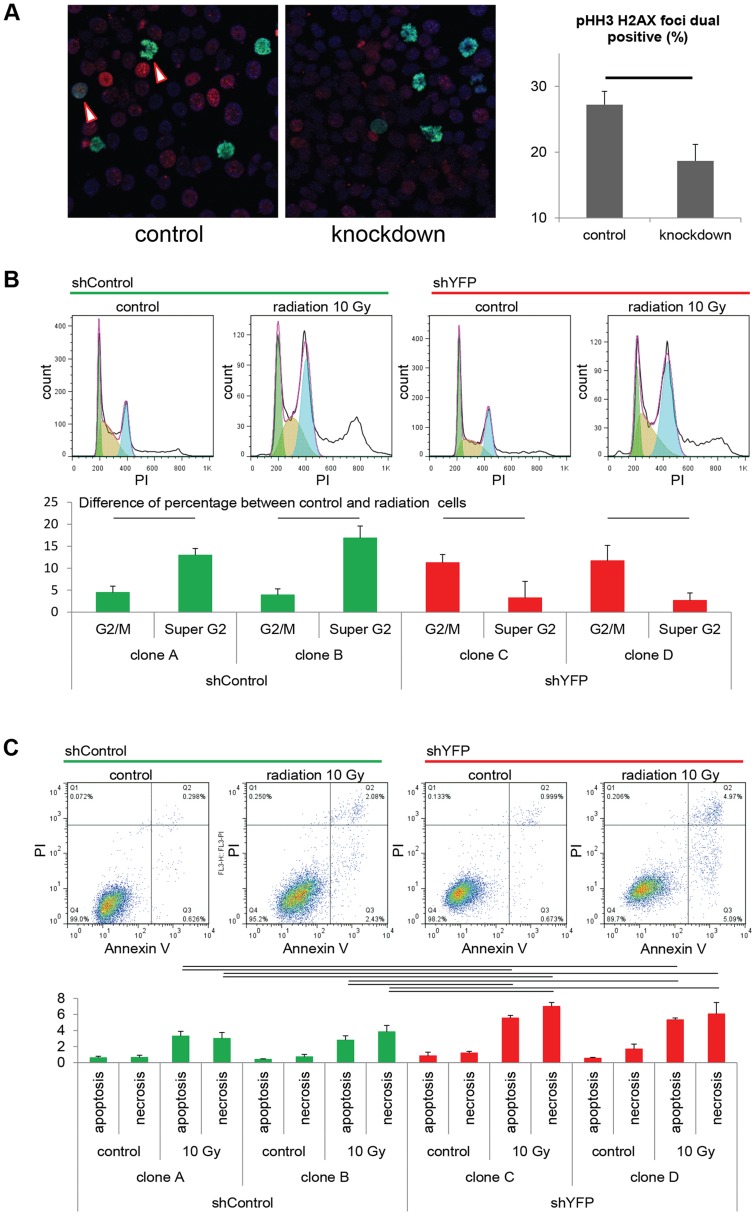
Pax3:Foxo1a facilitates G_2_/M checkpoint adaptation. (A) Immunocytochemistry for pHH3 (green), pH2AX (red) and DAPI (Blue) using U23674 mouse aRMS primary cell culture with or without *Pax3:Foxo1a* knockdown treated with 6 Gy irradiation. Black line shows significant difference (p<0.05). See Figure S2A for representative single-channel ICC images corresponding to Figure 5A. Arrowheads indicate pHH3 and pH2AX double positive cells. (B) Representative cell cycle analysis for U23674 transfected by shControl (Clone A) or shYFP (Clone C) with or without 10 Gy irradiation. The graph shows the differences of percentage between control and radiated cells in shControl and shYFP clones in 3 independent experiments. Black line shows significant difference (p<0.05). (C) Annexin V apoptosis detection assay for U23674 transfected by shControl or shYFP clones with or without 10 Gy irradiation. Black line shows significant difference (p<0.05).

As a test of checkpoint adaptation and the permissiveness of aRMS cells to transit from G_2_ to mitosis despite single- and double-stranded DNA damage, we irradiated tumor cells with or without *Pax3:Foxo1a* knockdown. Radiation resulted in a higher fraction of DNA breaks amongst mitotic cells (as represented by dual pHH3 positive, H2AX positive cells) under conditions of Pax3:Foxo1a expression than its knockdown (Figure 5A and Figure S2A), suggesting that Pax3:Foxo1a does facilitate G_2_ to M transition, consistent with checkpoint adaptation. Moreover, we performed cell cycle and Annexin V apoptosis detection assay after treatment with 10 Gy radiation for two independent eYFP shRNA knockdown clones compared to two other independent shRNA controls (as stated early, eYFP knockdown also achieves Pax3:Foxo1a knockdown) (Figure S2). Cell cycle analysis of the shRNA clones treated with radiation revealed increasing percentage of cells in cells having ≥4N DNA content after radiation for *Pax3:Foxo1a* knockdown cells compared to radiated controls (p<0.05)(Figure 5B). This result is consistent with a role of Pax3:Foxo1a in overcoming G_2_ arrest or M checkpoint arrest after radiation. Similarly, the Annexin V apoptosis detection assay showed a lower induction of apoptosis following radiation when Pax3:Foxo1a expression was preserved in shControl clones than shYFP cells (Figure 5C).

**Figure 6:**
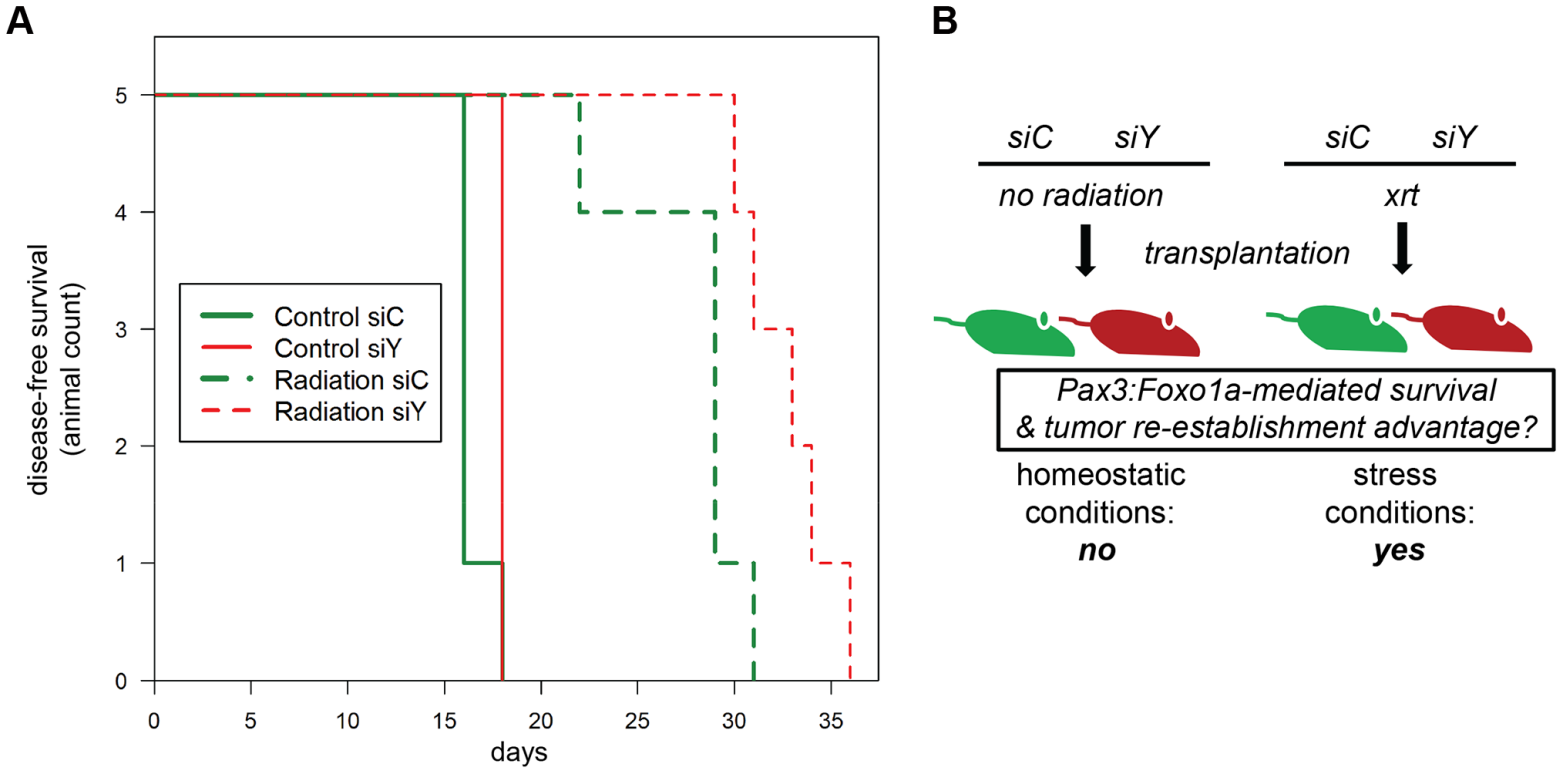
Treatment-related implications for dynamic oncogene expression in rhabdomyosarcoma *in vivo*. (A) Kaplan-Meier survival analysis for disease-free survival of mice implanted with pre-irradiated (10Gy) primary murine aRMS tumor cells treated with *Pax3:Foxo1a* siRNA (siY) or control siRNA (siC), *n* = 5 animals per cohort. The p value for the difference between siY and siC groups receiving radiation was 0.02. (B) Diagrammatic representation of results in (A).

To test the acute role of Pax3:Foxo1a in tolerization to treatment-related DNA damage *in vivo*, we used eYFP siRNA to transiently knock down Pax3:Foxo1a in aRMS tumor cells treated with radiation versus non-irradiated controls that were then orthotopically injected into unirradiated host mice. Pax3:Foxo1a mediated a cell survival and tumor re-establishment advantage under the stress condition of irradiation, but not under homeostatic conditions (p = 0.02, Figure 6A and 6B).

To assess the extent to which the fusion gene mediates refractoriness to chemotherapy agents, we observed Pax3:Foxo1a to facilitate 2–4 fold refractoriness to clinical agents capable of causing double-stranded DNA breaks and mitotic arrest (vincristine, actinomycin-D, topotecan) more so than agent inducing single-strand breaks (mafosfamide, the active metabolite of cyclophosphamide) (Figure S3A–E). That a similar role of Pax3:Foxo1a may apply to targeted agents was previously suggested by enriched G_2_ expression of Pdgfra (Figure 2F) and then demonstrated by increased sensitivity to prototypic Pdgfr inhibitor, imatinib, after *Pax3:Foxo1a* knockdown (Figure S3F). Similarly, *Pax3:Foxo1a* knockdown sensitized tumor cells to siRNA inhibition of downstream signaling mediators of acquired imatinib resistance (Figure S3G) [17]. Thus, these *in vitro* and *in vivo* results are consistent with a function of Pax3:Foxo1a in mediating checkpoint adaptation and refractoriness to the established clinical therapies of radiation and chemotherapy, or more contemporary molecularly-targeted agents.

## Discussion

A key finding of this study is that Pax3:Foxo1a expression is dynamic and varies during the cell cycle. To our knowledge this is first report of a translocation-mediated chimeric transcription factor oncogene that is expressed in a cell cycle-specific manner – much less, one that is expressed specifically during G_2_. The master transcription factor MYOD is expressed strongly during G_1_ [18] but is inactivated by phosphorylation during mitosis, which results in deportation from the nucleus [19]. MYF5 is also expressed in a cell cycle-dependent manner, but neither MYOD nor MYF5 expression is increased during G_2_/M as observed in our study of Pax3:Foxo1a in aRMS. Our findings reveal that *Pax3* expression in wildtype C2C12 myoblasts is dynamic and increased during G_2_/M, but that to account for the dramatic increase in *Pax3:Foxo1a* expression an additional enhancer effect of *Foxo1a* 3′ region DNA is likely to be present. This result opens the possibility that co-factors assembled at the *Pax3* promoter or fusion gene specific *cis*-elements might be targeted to suppress Pax3:Foxo1a expression.

Cell cycle progression after DNA damage is regulated by checkpoint controls, which prevent continued transit through the cycle until the damage has been repaired, hence protecting the integrity of the genome. Arrest in G_1_ permits repair prior to replication, whereas arrest in G_2_ allows repair prior to mitotic chromosome segregation. The p53 tumor suppressor, which is mutated in roughly half of human aRMS, has been shown to be integral to both G_1_ and G_2_ damage checkpoint machinery, but some reports found p53 dispensable for the G_2_ checkpoint [13], [20].

Checkpoint adaptation is defined as the ability to divide and survive following a sustained checkpoint arrest despite the presence of unrepairable DNA breaks [14]. Cells undergoing checkpoint adaptation will frequently die in subsequent cell cycles if DNA damage goes unrepaired, yet, some cells may be able to survive and proliferate in an aneuploid state [14]. Furthermore, in unicellular eukaryotes and tumor cells, DNA repair can occur at G_1_ [21]. Here, we reveal that the G_2_/M adaptation genes (*H2afx*, *Cdc25b* and *Plk1*) were suppressed by *Pax3:Foxo1a* knockdown in G_2_ and M cell cycle phases and that fewer cells transited from G_2_ to M without initiating apoptosis under conditions of *Pax3:Foxo1a* knockdown in the context of radiation-induced stress. These results suggested that not only cell cycle dependent expression but also a clinically-relevant biology underlying Pax3:Foxo1a expression at the G_2_-M checkpoint, a critical cell cycle checkpoint following radiation or DNA double strand break inducing-chemotherapy.

That a myogenic cancer might utilize genomic instability, aneuploidy or multinucleation as a mechanism of cell survival or tumor cell evolution/progression may not be so unexpected, in retrospect. Normal myofibers are typically multi-nuclear by definition, and genetic conditions predisposing to mitotic disjunction such as Mosaic Variegated Aneuploidy (MVA) are strongly associated with the development of RMS [22]. Both aRMS and eRMS have also been documented to be hyperdiploid, tetraploid, polyploid or to even have mixed aneuploid populations [23], [24], [25]. At a cellular level, the heterogeneity of cells in rhabdomyosarcoma is notable for the subpopulation of multi-nucleated rhabdomyoblasts which appears with giant nuclei or as multi-nucleated giant cells, often with cross-striations – yet highly mitotic [26]. These rhabdomyoblasts might be compared to the multinucleated stemloid cells in fibrosarcoma, which have a tumor-repopulating ability [27]. Our recent study of aRMS and the PKC iota inhibitor, aurothiomalate, reveals that aRMS cells have a remarkable tolerance to polyploidy, which induces neither apoptosis or senescence [28]. This intrinsic capacity to tolerate aneuploidy as well as this report's observed Pax3:Foxo1a-mediated increase in checkpoint adaptation gene expression may be directly relevant to clinical care, given that decreased expression of these same factors (*i.e.*, PLK1, CCCNB1, BIRC5, AURKB) have been reported to improve sensitivity to mitotic inhibitors [29]. Therefore, the interest generated from chemical screens identifying PLK1 as a potential therapeutic target in RMS [30] is likely warranted.

When considering the differences in treatment-related outcomes in RMS subtypes, the role of Pax3:Fox01a in checkpoint adaptation may be our most important clue yet as to how to improve outcome for fusion positive patients: while aRMS are certainly sensitive to standard chemotherapy and radiation, it is the survival of resistant clones which is the cause of disease progression and relapse – which occur to a greater extent in Pax:Foxo1a positive aRMS than fusion negative aRMS or eRMS [31], [32], and which we believe to be a result of *Pax3:Foxo1a*-mediated checkpoint adaptation. These effects on tumor cell sensitivity to radiation, chemotherapy and targeted therapeutics are likely to be cumulative and possibly critically important in defining the otherwise very narrow therapeutic window for fusion positive aRMS, for which the toxicity of chemotherapy and radiation is now dose-limiting [33].

Perhaps the most interesting aspect of this genetically-engineered conditional mouse model of a deadly but rare childhood cancer is that a labor-intensive knock-in approach to modeling the molecular pathophysiology of a fusion gene was beneficial. Successful transgenic tumor models have been generated by constitutive, ectopic expression of translocation-related fusion oncogenes for synovial sarcoma [34] as well as other “driver” oncogene related tumors [35]; similarly, retroviral transfection of oncogenes into hematopoietic cells has enabled this study of translocation-associated leukemia for many years [36], [37]. However, are these systems driven by non-native or partial-native promoters to be the definitive preclinical platforms for interrogating molecular physiology – or are distal native *cis*- and *trans*-regulation temporally critical? Every experimental system has its advantages and limitations, yet for cell and animal models where translocation-mediated fusion genes have yet to be modeled at the native promoter, we may have an entirely new spectrum of cancer genetics to explore.

## Materials and Methods

## Ethics statement

All animal procedures were conducted in accordance with the Guidelines for the Care and Use of Laboratory Animals and were approved by the Institutional Animal Care and Use Committee (IACUC) at the University of Oregon Health & Science University (OHSU) or the Joslin Diabetes Center (Boston, MA). Every effort was made to minimize suffering.

### Mice

The *Myf6Cre,Pax3:Foxo1a,p53* conditional aRMS mouse model has been described previously [5], [6], [7], is described as caMOD Model 150064393, and is publically available through the NCI MMHCC Repository (MMHCC Strain Codes 01XBL B6; 129-Myf6<tm2(Cre)Mrc> and 01XBM B6; 129-Pax3<tm1Mrc>). SHO-*Prkdc^scid^Hr^hr^* mice were purchased from Charles River Laboratories (Wilmington, MA) and bred/maintained at OHSU.

### Primary tumor cell cultures and cell lines

Mouse primary cell cultures (U23674, U42369, U57844) were established from tumor samples. Tumors were minced into small pieces and digested with collagenase (10 mg/ml) overnight at 37°C. The dissociated cells were then incubated in Dulbecco's modified eagle's media supplemented with 10% fetal bovine serum (FBS) and 1% penicillin-streptomycin in 5% CO_2_ at 37°C. C2C12 mouse myoblast cells were purchased from ATCC (Manassas, VA). Human aRMS cell lines were a gift from Peter Houghton (Rh3; Nationwide Children's Hospital, Columbus, OH) or Patrick Reynolds (Rh41; COG Cell Culture and Xenograft Repository). These cells lines were maintained in the same culture conditions as primary tumor cell cultures: DMEM supplemented with 10% Fetal Bovine Serum (FBS) and 1% Penicillin-Streptomycin. All primary cell cultures experiments using cells were carried out at passage 3–7.

### Confocal imaging

For immunofluorescence staining of frozen sections, the polyclonal antibody for green fluorescent protein (1∶1000, AB16901, Chemicon) was used with DAPI counterstain.

### RNA interference studies

siRNA transfections were carried out using Lipofectamine2000 (Invitrogen, Grand Island, NY) according to manufacturer's recommended protocol. siRNA's were diluted between 0.1 and 10 nM, and the final concentration of Lipofectamine2000 was 0.2%. siYFP Stealth RNAi siRNA Reporter Controls (cat. 12935-145; Invitrogen) were used as the eYFP siRNA to knockdown the *Pax3:Foxo1a-ires-* *eYFP* bi-cistronic mRNA, whereas Stealth RNAi siRNA Negative Control Med GC #3 (cat. 12935-113; Invitrogen) was used as the siRNA control (siCont).

### Generation of shRNA tumor cell culture clones

To establish shRNA knockdown clones of primary tumor cell cultures, we used MISSION pLKO.1-puro eGFP shRNA Control Transduction Particles (cat. SHC005V; Sigma Aldrich) for Pax3:Foxo1a knockdown and MISSION pLKO.1-puro Non-Mammalian shRNA Control Transduction Particles (cat. SHC002V; Sigma Aldrich) as the control, respectively. shRNA transfections and clonal selection were carried out according to manufacturer's recommended procedures. Mouse RMS primary cell cultures were plated at 1.8×10^6^ cells per 150 mm dish. After 24 h, hexadimethrine bromide was added (8 µg/ml, cat. H9268; Sigma Aldrich), followed by each particle solution (MOI 0.5). After another 24 h, media were removed and fresh media were added. The following day, puromycin was added (5 µg/ml, cat. P8833; Sigma Aldrich). Puromycin-resistant clones were selected cloning rings at day 14 (shControl) and day 17 (shYFP), with continuous puromycin selection at all times.

### Radiation

Cells were irradiated on a Trilogy linear accelerator (Varian, Palo Alto, CA) with a 10×10 cm AP field. Two centimeter of bolus material was placed on top of the 2 chamber slide or 6 cm dish and the target surface distance to the bolus was at 97 cm. Monitor units on the linear accelerator were then set to deliver 6 Gy or 10 Gy of dose to the cells.

### Immunoblotting

Tumors were lysed in radioimmunoprecipitation assay (RIPA) buffer or NP40 buffer containing both protease and phosphatase inhibitor (Sigma). The lysates were homogenized and centrifuged at 8000 g for 10 minutes. The resulting supernatants were used for immunoblot analysis. Goat anti-FOXO1A antibody (cat. Sc-9808; Santa Cruz, Santa Cruz, CA), goat anti-GFP antibody (cat. 600-101-215, Rockland; Gilbertsville, PA) or rabbit anti-PDGFRa antibody (cat. #3164; Cell signaling Technology, Danvers, MA).

### Immunocytochemistry

Cells were plated on 8-well CultureSlides (cat. 354118; BD Falcon, Franklin Lakes, NJ), fixed with 4% paraformaldehyde, permeabilized with 0.1% or 0.25% TritonX100, washed and incubated with mouse monoclonal anti-skeletal myosin (FAST) (cat. M4276; Sigma), rabbit anti-Ki67 (cat. RM-9106-F; Thermo Scientific, Waltham, MA), mouse anti-Pax3 (cat. MAB2457; R&D Systems), mouse anti-phospho Histone H3 (cat. #9706; Cell Signaling Technology), rabbit anti-phospho Histone H3 (cat. #3377; Cell Signaling Technology), mouse anti-phospho Histone H3 (cat. #9706; Cell Signaling Technology), rabbit anti-CDC2-Y15 (cat. #4539; Cell Signaling Technology) or rabbit anti-phospho H2AX antibody (cat. #9718; Cell Signaling Technology), overnight, rinsed with PBS, incubated with fluorescein isothiocyanate-conjugated anti-mouse and rabbit IgG (1∶200) for 1 h, and examined by confocal microscopy with a Zeiss LSM700 instrument. For immunocytochemistry experiments, at least 100 positive cells were scored per specimen.

### FACS sorting

Cells were suspended in Hank's balanced salt solution (HBSS) with 2% FBS and 2 mM EDTA. Antibody staining was performed for 20 minutes on ice. Prior to FACS sorting, cells were suspended in 1 µg/ml propidium iodide (Pi) and 10 µM calcein blue (Invitrogen) to identify viable cells (Pi^−^Ca^+^). Purity checks were performed to confirm that the sorted eYFP+ and eYFP- cell subsets had a purity of >98% using a eYFP expression threshold determined by the background fluorescence of eYFP- C2C12 cells. The following antibodies were used to evaluate receptor tyrosine kinase surface expression: APC-conjugated Pdgfrα antibody (#17-1401-81, eBiosciences) or anti-IGF1 Receptor antibody (cat. Ab32823; Abcam, Cambridge, MA; 1 in 25).

### Cell cycle analysis

Mouse RMS primary cell cultures were trypsinized and incubated with Hoechst33342 (final concentration 15 µg/ml) and Reserpine (final concentration 5 µM). Cells were incubated in the dark for 30 min at 37°C, and analyzed and sorted by flow cytometry using an Influx FACS instrument (Becton Dickinson, Franklin Lakes, NJ). Cell cycle was determined with the FlowJo software (Tree Star, Inc., Ashland, OR).

### Annexin V apoptosis detection assay

Mouse primary cell cultures were stained with Annexin V and Propidium iodide using Annexin-V-FLUOS Staining Kit (cat. 11 858 777 001; Roche) following the protocol provided by the manufacturer. Briefly, 48 hour after irradiation, 10^6^ mouse primary cell cultures were trypsinized, washed by PBS and resuspended in 100 µl of Annexin-VFLUOS labeling solution, incubated 10–15 min at 15–25°C, and analyzed by FACS Calibur.

### Quantitative RT PCR (QPCR)

U23674 cells were subfractionated by FACS sorting as described above. mRNA was isolated using RNeasy spin columns (Qiagen, Valencia, CA) and reverse transcribed using Superscript III First-Strand Synthesis System for RT-PCR (Invitrogen). QPCR was performed using an AV7900 PCR system (Applied Biosystem) with SYBR-green PCR reagents. *Pax3:Foxo1a* was detected using the following primer sequences: 5′-*AGACAGCTTTGTGCCTCCAT*-3′ and 5′-*CTCTTGCCTCCCTCTGGATT*-3′. Other primers are Taqman Gene Expression assay, *H2afx* (Mm00515990_s1), *Cdc25b* (Mm00499136_m1), *Birc5* (Mm00599749_m1), *Plk1* (Mm00440924_g1) and *Gapdh* (Mm99999915_g1) by Invitrogen. RT-PCR data were quantified using the standard curve method, and relative expression of *Pax3:Foxo1a* per sample was determined by normalization against the quantity of 18 s rRNA and *Gapdh* within each sample. For each sample, QPCR was performed in technical duplicates and results were averaged.

### *In vitro* growth inhibition assays

Mouse RMS primary cell cultures were plated at 1×10^3^ cells of each cohort per well in a 96-well plate. After cell incubations, cytotoxic effects were assayed using CellTiter 96 AQueous One Solution Cell Proliferation Assay system (Promega, Madison, WI) and SpectraMax M5 luminometer (Molecular Devices, Sunnyvale, CA). IC50 and C.I. were determined with CalcuSyn software (BIOSOFT, United Kingdom).Drugs: Vincristine sulfate salt (cat. V8879; Sigma), Actinomycin-D (cat. A9415; Sigma), Mafosfamide (cat. sc-211761; Santa Cruz), Topotecan hydrochloride (cat. S1231; Selleck), Eribulin mesylate (NDC 62856-389-01; Eisai) or Imatinib Mesylate (cat. S1026; Selleck).

### RNAi-assisted protein target identification (RAPID) screen

For these studies, individual siRNA were obtained from Dharmacon (Lafayette, CO), including the mouse siRNA library targeting the tyrosine kinome (siGENOME). These experiments are performed at 100 nM concentration and include non-specific pooled siRNA as a control purchased from Dharmacon. Transfection of siRNA was carried out using Lipofectamine 2000 in Opti-MEM Reduced Serum Media (Invitrogen). After cells were plated in 96-well plates in the presence of inhibitor or siRNA, and incubated for 96 hours, respectively, 20 µL CellTiter 96 AQueous One solution (MTS) was added to each well and absorbance values assessed by the BioTek Synergy 2 plate reader (BioTek, Winooski, VT).

### Genome-wide expression analysis

Labeled target cRNA was prepared from 12 mouse total RNA samples (3 independent experiments×4 samples). Samples were amplified and labeled using the Ambion MessageAmp Premier RNA Amplification Kit following the manufacturer's protocol. Sample order was randomized. Each sample target was hybridized to an Illumina MouseRef 8 v 2 Expression BeadChip Array. Image processing and expression analysis were performed using Illumina BeadArray Reader and GenomeStudio (v. 2010.1) Gene Expression module (v. 1.6.0) software. Microarray data have been accessioned with the Gene Expression Omnibus (GEO) under series GSE41675. The following link has been created to allow review of record GSE41675 while it remains in in review/under private status: http://www.ncbi.nlm.nih.gov/geo/query/acc.cgi?token=xdajbqeisomcyhq&acc=GSE41675.

### *In vivo* studies with *Pax3:Foxo1a* knockdown and radiation

aRMS primary cultures (passage 5) were plated in 6 cm dishes. The next day cells were transfected with siYFP Stealth RNAi siRNA Reporter Controls or Stealth RNAi siRNA Negative Control Med GC #3. Two days later cells were irradiated on a Trilogy linear accelerator with a 10×10 cm AP field with two centimeter of bolus material was placed on top of the 6 cm dish. The target surface distance to the bolus was at 97 cm and monitor units on the linear accelerator were then set to deliver 10 Gy of dose to the cells. Subsequently, cells were trypsinized and 500,000 cells were injected into the gastrocnemius muscle of SHO mice that had been pre-injured 24 hours prior with 0.85 µg/mouse cardiotoxin intramuscularly. Tumor volumes (cm^3^) were measured 3-dimensionally with electronic calipers and calculated from formula (π/6)×length×width×height, assuming tumors to be spheroid. For statistical analysis of disease-free survival, a tumor volume threshold of 0.25 cc was applied. The log-rank test was used to contrast treatments. All analyses were performed using R 3.0.0 (The R Foundation for Statistical Computing, Vienna, Austria).

## Supporting Information

Figure S1 *This supplemental figure relates to* *Figure 1*. eYFP activity and Pax3:Foxo1a expression is dynamic. (A) eYFP fluorescence of eYFP sorted U42369 mouse aRMS primary cell culture overtime as measured by FACS. Grey: C2C12 (negative control), blue: no sorted cells, green: eYFP activity high cells, red: eYFP activity low cells. (B) Mean of relative eYFP activity measured by FACS. (C) Western blot analysis using eYFP sorted cells. Plotted are relative protein levels of Pax3:Foxo1a/β-actin. Mean ± SE were obtained from three independent immunoblottings. Black line shows significant difference (p<0.05). (D) eYFP activity and cell cycle analysis using Hochest33342 staining for mouse primary cell culture U42369. Green shows G_0_/G_1_ phase, brown shows S phase, and blue shows G_2_/M phase. (E–F) Proliferating mouse aRMS tumor cells were treated with 10 µg/ml CHX for the indicated incubations, and eYFP, Pax3:Foxo1a and Pdgfra protein levels were followed by western blot analysis (E). Protein expression quantified as relative flux normalized by β-actin for calculation of protein half-lives (F).
(TIF)

Figure S2 *This supplemental figure relates to* *Figure 5*. Pax3:Foxo1a mediates checkpoint adaptation. (A) Individual and merged channels for immunocytochemistry of pHH3 (green), pH2AX (red) and DAPI (blue) using U23674 mouse aRMS primary cell culture with or without *Pax3:Foxo1a* knockdown treated by 6 Gy irradiation. (B) Western blot analysis of Pax3:Foxo1a and Foxo1a in U23674 shControl and shYFP clones.
(TIF)

Figure S3 *This supplemental figure relates to* *Figure 6*. Pax3:Foxo1a modifies the aRMS therapeutic response and remains an essential target. (A–E) *Pax3:Foxo1a* knockdown increases select chemotherapy sensitivities. MTS assay was performed for *Pax3:Foxo1a* knockdown mouse aRMS tumor cells treated with DNA damaging agents and microtubule inhibitors. *Pax3:Foxo1a* knockdown reduced the concentration at which viability was impaired by 50% (IC50) of vincristine, actinomycin-D, topotecan and eribulin by 2.9, 3.4, 4.8 and1.8 fold, respectively, yet did not affect the IC50 of mafosfamide. (F–G) Imatinib IC50 determination using mouse aRMS tumor cells transfected with siCont or siYFP, respectively. *Pax3:Foxo1a* knockdown sensitized aRMS cells 5-fold to this prototypic Pdgfra inhibitor. Given the role of Pax3:Foxo1a in growth factor receptor transcription, we next explored the role of Pax3:Foxo1a in driving aberrant tyrosine kinase signaling by means of an RNAi-assisted protein target identification (RAPID) screen after first knocking down *Pax3:Foxo1a* in mouse aRMS tumor cells [38]. Cell viability was significantly decreased not only for targets of imatinib but also for mediators of imatinib resistance [17] in *Pax3:Foxo1a* knockdown cells compared with control cells (G). * p<0.01.
(TIF)

Movie S1 *This supplemental movie relates to* *Figure 1*. Time lapse of eYFP expression in murine aRMS cells.
(MOV)

Table S1 Genome-wide expression analysis using cell cycle specific sorted cells (2N vs 4N) with or without Pax3:Foxo1a knockdown.
(XLSX)

Table S2 Putative Pax3:Foxo1a binding sites of Checkpoint Adaptation related genes.
(XLSX)
