# Supplementary figures and images for: Cell-Cycle Dependent Expression of a Translocation-Mediated Fusion Oncogene Mediates Checkpoint Adaptation in Rhabdomyosarcoma
Source: PLoS Genet. 2014 Jan 16;10(1):e1004107. doi: 10.1371/journal.pgen.1004107 (PMC3894165; doi:10.1371/journal.pgen.1004107)

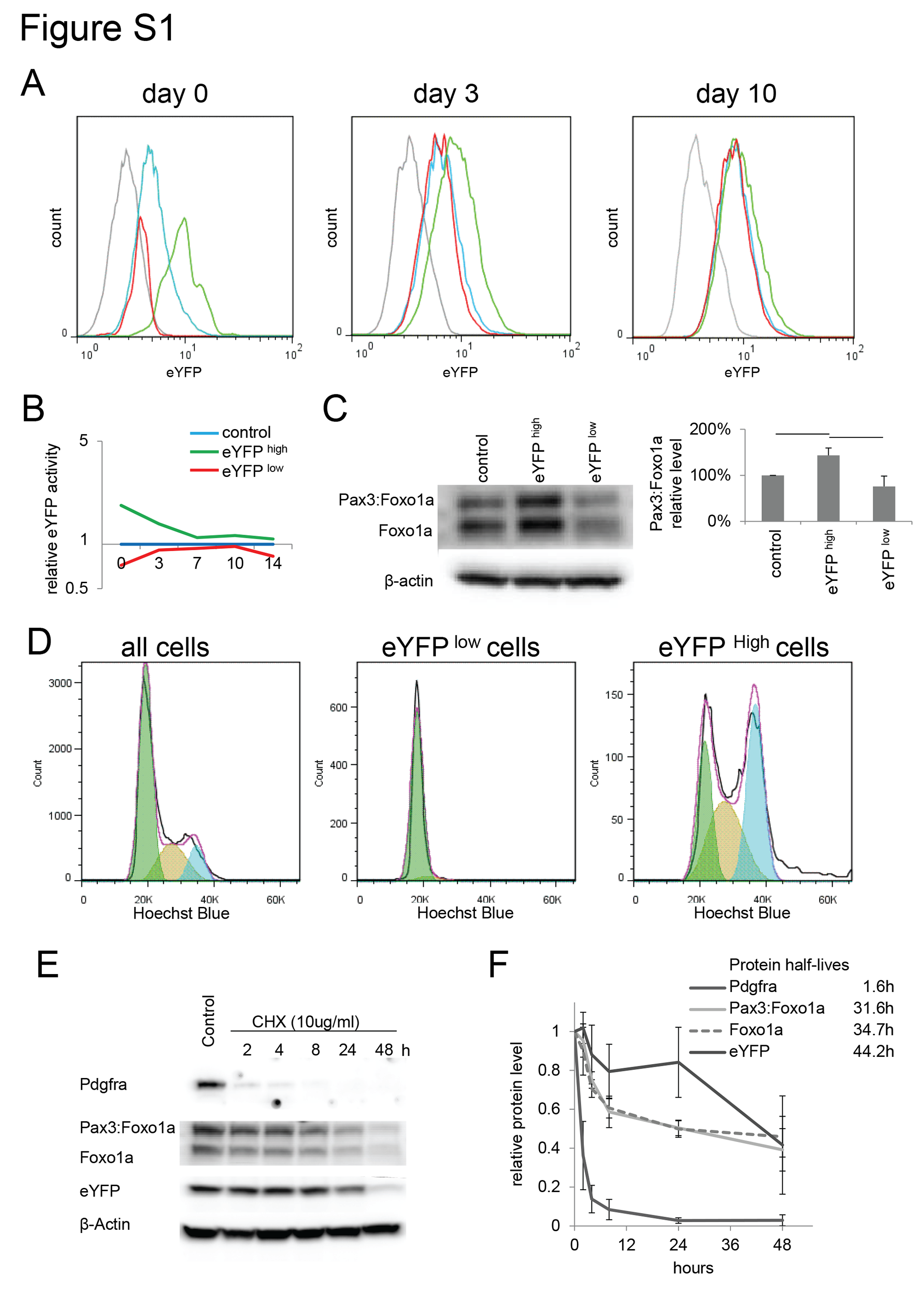

Supplement: Figure S1 — This supplemental figure relates to Figure 1 . eYFP activity and Pax3:Foxo1a expression is dynamic. (A) eYFP fluorescence of eYFP sorted U42369 mouse aRMS primary cell culture overtime as measured by FACS. Grey: C2C12 (negative control), blue: no sorted cells, green: eYFP activity high cells, red: eYFP activity low cells. (B) Mean of relative eYFP activity measured by FACS. (C) Western blot analysis using eYFP sorted cells. Plotted are relative protein levels of Pax3:Foxo1a/β-actin. Mean ± SE were obtained from three independent immunoblottings. Black line shows significant difference (p<0.05). (D) eYFP activity and cell cycle analysis using Hochest33342 staining for mouse primary cell culture U42369. Green shows G0/G1 phase, brown shows S phase, and blue shows G2/M phase. (E–F) Proliferating mouse aRMS tumor cells were treated with 10 µg/ml CHX for the indicated incubations, and eYFP, Pax3:Foxo1a and Pdgfra protein levels were followed by western blot analysis (E). Protein expression quantified as relative flux normalized by β-actin for calculation of protein half-lives (F). (TIF) [file pgen.1004107.s001.tif]

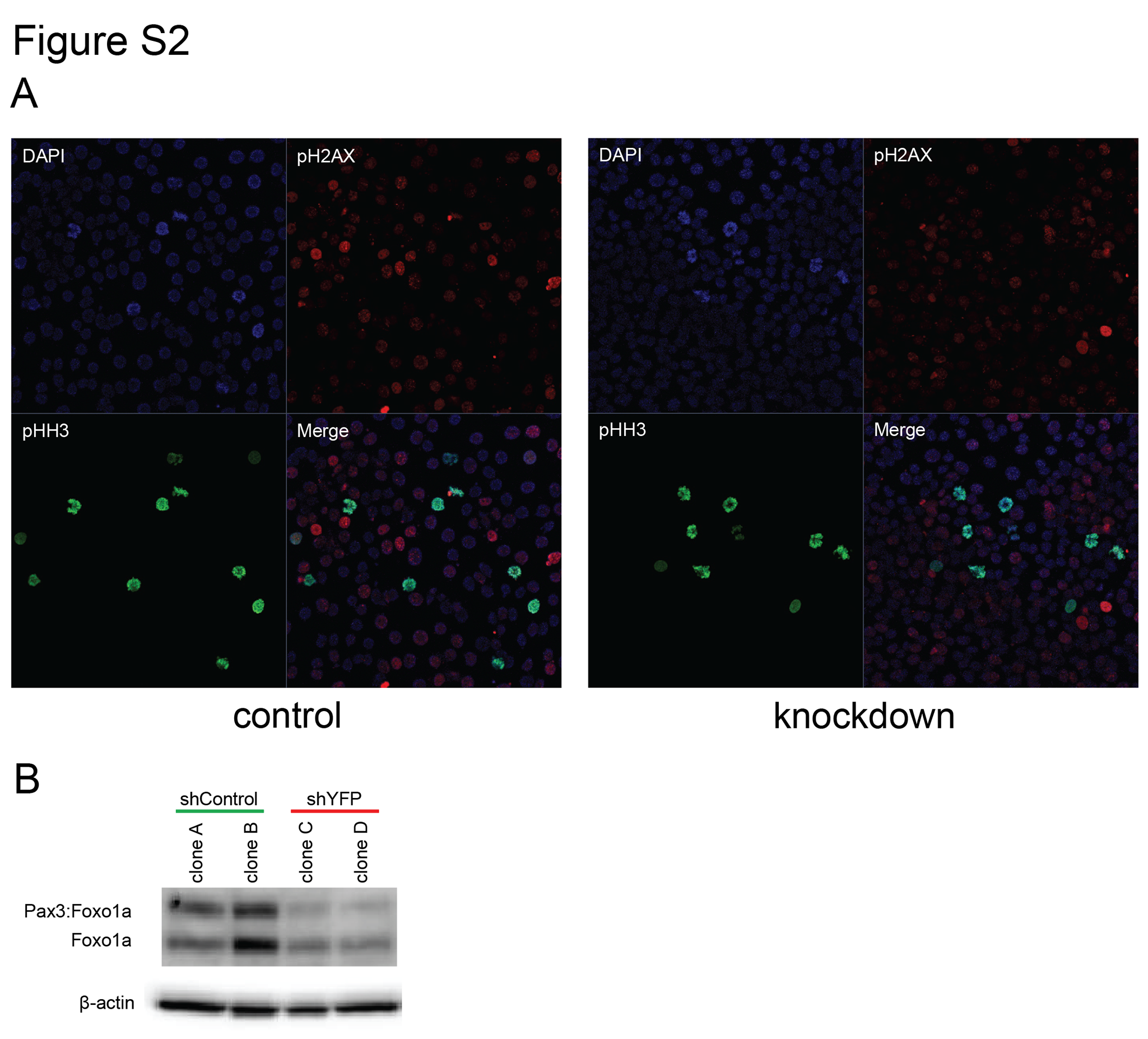

Supplement: Figure S2 — This supplemental figure relates to Figure 5 . Pax3:Foxo1a mediates checkpoint adaptation. (A) Individual and merged channels for immunocytochemistry of pHH3 (green), pH2AX (red) and DAPI (blue) using U23674 mouse aRMS primary cell culture with or without Pax3:Foxo1a knockdown treated by 6 Gy irradiation. (B) Western blot analysis of Pax3:Foxo1a and Foxo1a in U23674 shControl and shYFP clones. (TIF) [file pgen.1004107.s002.tif]

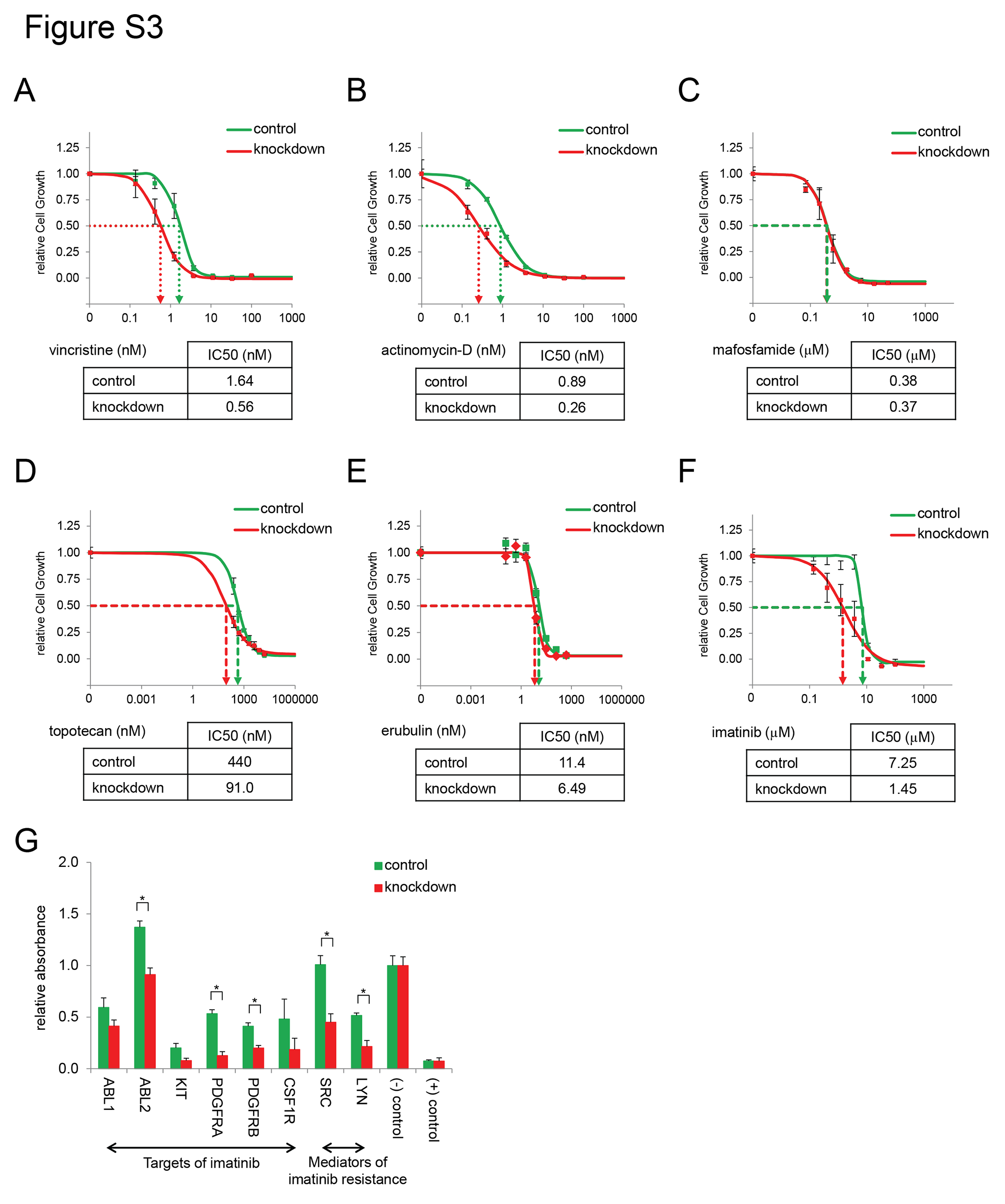

Supplement: Figure S3 — This supplemental figure relates to Figure 6 . Pax3:Foxo1a modifies the aRMS therapeutic response and remains an essential target. (A–E) Pax3:Foxo1a knockdown increases select chemotherapy sensitivities. MTS assay was performed for Pax3:Foxo1a knockdown mouse aRMS tumor cells treated with DNA damaging agents and microtubule inhibitors. Pax3:Foxo1a knockdown reduced the concentration at which viability was impaired by 50% (IC50) of vincristine, actinomycin-D, topotecan and eribulin by 2.9, 3.4, 4.8 and1.8 fold, respectively, yet did not affect the IC50 of mafosfamide. (F–G) Imatinib IC50 determination using mouse aRMS tumor cells transfected with siCont or siYFP, respectively. Pax3:Foxo1a knockdown sensitized aRMS cells 5-fold to this prototypic Pdgfra inhibitor. Given the role of Pax3:Foxo1a in growth factor receptor transcription, we next explored the role of Pax3:Foxo1a in driving aberrant tyrosine kinase signaling by means of an RNAi-assisted protein target identification (RAPID) screen after first knocking down Pax3:Foxo1a in mouse aRMS tumor cells [38]. Cell viability was significantly decreased not only for targets of imatinib but also for mediators of imatinib resistance [17] in Pax3:Foxo1a knockdown cells compared with control cells (G). * p<0.01. (TIF) [file pgen.1004107.s003.tif]
